# A Comparison of ACL Injury Risk, Ski Geometry and Standing Height Parameters between Skiers with Rented and with Owned Skis

**DOI:** 10.3390/ijerph191711124

**Published:** 2022-09-05

**Authors:** Gerhard Ruedl, Markus Posch, Katja Tecklenburg, Alois Schranz, Martin Faulhaber, Elena Pocecco, Martin Burtscher

**Affiliations:** 1 Department of Sport Science, University of Innsbruck, A-6020 Innsbruck, Austria; 2 Medalp Sportclinic, A-6460 Imst, Austria

**Keywords:** alpine skiing, ACL injury, rented skis, risk factors, standing height, ski widths, ski geometry

## Abstract

Aim: to evaluate if ACL injuries are associated with recreational skiers using rented skis and whether individual factors, ski geometry parameters and standing heights differ between skiers who rented or owned skis. A retrospective questionnaire-based, case–control study of ACL-injured and uninjured recreational skiers was conducted during six winter seasons. Age, sex, body height, body weight, nationality, ownership of skis, skill level, risk-taking behavior, ski length, side-cut radius, widths of the tip, waist, and tail, and the standing heights at the front and rear components of the ski binding were assessed. Additionally, ratios between ski widths and a standing height ratio were calculated. Altogether, 1780 skiers (48.9% females) with a mean age of 39.2 ± 13.0 years participated, of whom 22.0% sustained an ACL injury and 32.3% rented skis. ACL injury risk was significantly associated with rented skis (OR 3.2, 95% CI 2.5–4.0). Compared to skiers using own skis, participants who rented skis were more likely female, smaller and lighter, tourists, less skilled and more cautious. In comparison to owned skis, rented skis showed significantly lower mean values in ski length, side-cut radius, ski widths, and for the three ski widths ratios. Additionally, standing heights were significantly lower while standing height ratio was higher for rented skis. Beside individual factors, equipment-related factors should be considered when renting skis in order to reduce ACL injury risk.

## 1. Introduction

Recreational alpine skiing has become a very popular winter sport activity world-wide with hundreds of millions winter sport enthusiasts on ski slopes every year [1]. About 8 million skiers alone visit the Austrian Alps annually [2] consuming more than 50 million skier days [3]. Although recreational skiing impacts general health benefits related to physical activity [4] there is also an association with a certain risk of injury on Austrian ski slopes of less than 1 injury per 1000 skier days [5]. Despite the steady downward trend in the overall injury risk in recreational skiing in past decades, the knee joint is still the most affected anatomical location with about one third of all injuries [5,6]. Moreover, in approximately half of all serious knee injuries the anterior cruciate ligament (ACL) is affected [7,8].

**Figure 1:**
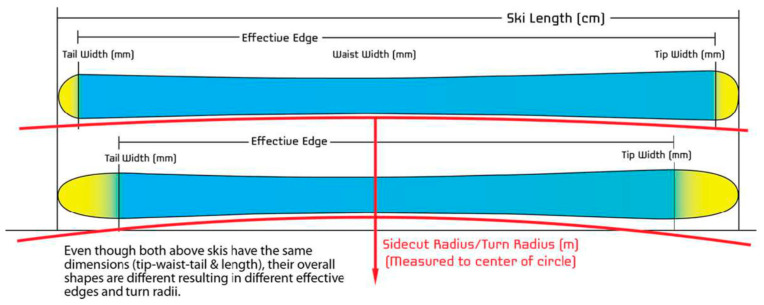
Ski geometry characteristics (ski length, side-cut radius, and widths of the tip, waist and tail of the ski). With permission from https://www.gearx.com/blog/knowledge/skiing/ski-shape-profile/, accessed on 2 September 2022.

There is evidence that a skiing-related injury is likely to be the result of a complex interaction of individual, equipment-related and environmental factors [1,9]. About 80% of lower extremity injuries, mostly including knee injuries, are equipment- related, caused by the ski acting as lever to bend or twist the leg [10]. Ski geometry parameters such as ski length, side-cut radius, widths of the tip, waist and tail of the skis (Figure 1) as well as standing heights seem to affect ACL injury risk in recreational skiing [11].

Due to its topography and skiing tradition, Austria is a popular country for winter sport tourists from foreign countries with about 50 million overnight stays yearly in winter months [12]. Thus, it is not astonishing that the number of rented skis in ski-rental shops is increasing, e.g., in the winter season 2018/19 about 3.7 million pair of skis were sold to sports retailers worldwide (which is an increase of about 17% compared to the previous season) and of those 60% of the skis were used for rental [13]. However, there seems to be evidence in the skiing literature that rented skis are associated with an increased injury risk [14,15]. Ekeland and Rodven [16] reported that 35% of injured skiers and snowboarders used rental equipment. Goulet et al. [17] showed in a study using a case–control design a more than 7-fold higher injury risk for children aged 12 years and younger when using rented equipment. Another case–control study on first-day skiers, snowboarders and skiboarders found that participants using own equipment were less likely to be injured than those using rented gear [18]. Aschauer et al. [19] evaluated potential risk factors for a ski injury in an Austrian ski region during six weeks in 2005. Regarding the impact of rented equipment on injury risk, they found a higher number of rented equipment in injured skiers as well as in injured snowboarders compared to the particular uninjured control group [19].

As there is evidence that ski equipment is affecting knee/ACL injury risk in recreational skiing [1,6,9,11], and due to the fact that about one third of injured skiers use rented skis [16], it seems important for the healthcare system as well as for ski manufacturers to evaluate the potential impact of ski geometry parameters of rented skis on knee/ACL injury risk.

To the best of our knowledge, there is no study investigating whether an ACL injury on ski slopes is associated with rented skis. In addition, there seems to be a lack of knowledge whether ski geometry parameters and standing heights differ between owned and rented skis. Thus, this study aimed to evaluate (a) if ACL injury risk is associated with rented skis, (b) potential differences in individual factors of people using own or rented skis, and (c) potential differences within ski geometry parameters and standing heights between owned and rented skis.

## 2. Materials and Methods

### 2.1. Participants

Inclusion criteria: uninjured and ACL-injured skiers > 17 years using any type of carving skis were interviewed by questionnaire during 6 consecutive winter seasons from 2014/15 to 2019/20 in a large ski area in the Western part of Austria [11]. Uninjured skiers were selected at different spots in the ski area throughout the whole skiing day. Only skiers, who injured their ACL after a fall without the involvement of other persons, were included and subsequently interviewed in a sports clinic, situated in this ski area. About 95% of all invited subjects agreed to participate in the study.

The study was performed in conformity with the ethical standards of the 2013 version of the Declaration of Helsinki. All included skiers were informed about the aims of the study and gave their written informed consent for participating. The survey was conducted according to the ethical guidelines for surveys approved by the Institutional Review Board (IRB) as well as the Board for Ethical Issues (BfEI) of the University of Innsbruck (certificate of good standing 29/2016). Analyses from this unique sample have already been reported [11].

### 2.2. Questionnaire

Participants were questioned using a hand-filled questionnaire on the intrinsic factors age, sex, body height and weight, and nationality (Austrian vs. others). In addition, skiers had to self-report their skiing skill level (expert, advanced, intermediate and beginner) [20] and risk-taking behavior (more risky vs. more cautious) [21]. Subsequently, participants were divided into more-skilled (expert and advanced) and into less-skilled (intermediate and beginner) skiers as a tendency was shown to underestimate individual skiing skills, especially among female skiers [20]. Additionally, ownership of ski equipment (own vs. rented in a ski shop vs. borrowed from family/friends) was asked. However, in this study only skiers who owned or rented their skis in a ski shop were included while skiers who borrowed the skis from family or friends (*n* = 37, no ACL injury) were excluded.

### 2.3. Ski Geometry Parameters and Standing Height

Ski length, side-cut radius as well as widths of the tip, waist and tail of the ski were directly notated from the carving ski of the subject. Furthermore, ski length was relativized by body height (relative ski length).

As in a recently published study [11] a wider ski tip increased the likelihood of an ACL injury while a wider ski waist decreased the likelihood of an ACL injury, ratios were built between the three widths of the ski. The ‘waist-to-tip width ratio’ was defined as the percentage between waist width and tip width of the ski, i.e., the lower this ratio the higher the skis’ tip (shovel) width in relation to the skis’ waist width. Similarly, the ‘waist-to-tail width ratio’ and the ‘tail-to-tip width ratio’ were defined as the percentage between the waist width and tail width, and between the tail width and tip width, respectively.

Standing height (i.e., the distance between the bottom of the running surface of the ski and the ski boot sole) at the front and rear component of the ski binding was measured using a digital sliding caliper and the percentage ratio between front and rear components of the ski binding was calculated according to Ruedl et al. [11].

Due to the nature of an ongoing study, the evaluation of various ski geometry parameters and standing heights were started at different timepoints. Thus, sample size can vary within single variables.

### 2.4. Statistics

Data are presented as means ± standard deviations and absolute or relative frequencies. After checking for normal distribution of metric data with the Shapiro–Wilk test, independent t-tests were used to compare normally distributed and Mann–Whitney-U tests for not normally distributed data of skiers who owned or rented their skis with regard to age; body height and weight; ski length; relative ski length; side-cut radius; widths of the tip, waist and tail of the ski; waist-to-tip width ratio, waist-to-tail width ratio, and tail-to-tip width ratio; and standing heights of the front and rear components of the ski binding, and standing height ratio.

In addition, differences in frequencies (ACL injury; sex; nationality; skiing skill level; and risk-taking behavior) were evaluated using Chi-square tests.

A univariate odds ratio (OR) with a 95% confidence interval was calculated to investigate the association between ACL injury and rented skis.

SPSS 26.0 (IBM Corporation, Armonk, NY, USA) was used for the statistical analysis. All *p*-values were two-tailed and values below 0.05 were considered to indicate statistical significance.

## 3. Results

A total of 1780 recreational skiers (48.9% females) were included in this study, of whom 78.0% were uninjured and 22.0% sustained an ACL injury. The mean age, height, and weight for the total cohort was 39.2 ± 13.0 years, 173.8 ± 8.7 cm, and 74.7 ± 13.8 kg, respectively. Regarding nationality, 31.0% were Austrians, 71.7% of skiers self-reported their ability to be “more skilled” and 34.0% of participants reported a “riskier behavior” during skiing. A total of 575 (32.3%) rented skis in a professional ski-rental shop.

Mean absolute ski length, relative ski length, side-cut radius, tip width, waist width and tail width of the total cohort was 164.7 ± 9.7 cm, 94.8 ± 3.8%, 14.4 ± 2.7 m, 121.0 ± 8.0 mm, 75.1 ± 8.5 mm, and 106.6 ± 7.9 mm, respectively.

Mean waist-to-tip width ratio, mean waist-to-tail width ratio, and mean tail-to-tip width ratio of the total cohort was 63.0 ± 6.1%, 71.5 ± 6.3%, and 88.4 ± 7.4%, respectively.

Mean standing height at the front component of the ski binding, standing height at the rear component of the ski binding, and the standing height ratio was 39.9 ± 5.6 mm, 44.6 ± 6.2 mm, and 89.7 ± 6.9%, respectively.

Table 1 shows the univariate comparison of included factors between skiers who owned or rented their skis. With the exception of age, all other factors significantly differed between the two groups. ACL injury risk was significantly associated with rented skis (OR 3.2, 95% CI 2.5–4.0, *p* < 0.001).

## 4. Discussion

The aim of this study was to evaluate if an ACL injury is associated with rented skis and whether individual factors and/or ski geometry parameters and standing heights differ between skiers who owned or rented skis. We found a significant association between an ACL injury and rented skis. In addition, skiers who rented skis were more likely female, smaller and lighter, tourists from outside of Austria, lower-skilled and more cautious skiers. Ski length, side-cut radius and ski widths showed significantly lower values for rented skis as well as for the three ski width ratios. Standing heights of the front and rear components of the ski binding were significantly lower while standing height ratio was significantly higher for rented skis.

### 4.1. ACL Injury, Sex, Anthropometrics, Nationality, Skill Level and Risk-Taking Behavior

About one third of study participants rented their skis. In accordance, Ekeland et al. [22] reported for alpine skiing a use of rented skis of 34% in children and 32% in adults.

ACL injury risk is multiplied by more than 3-fold when using rented skis in this study. In comparison, Bouter et al. [23] found in a case–control study with more than 1000 skiers that the use of rented or borrowed skis was associated with an about 2-fold higher risk for an injury of the lower extremity. Moreover, Hume et al. [15] reported in a meta-analysis for skiers/snowboarders who rent snow equipment an odds ratio of 2.6 for sustaining a snow-sport injury. Interestingly, Hume et al. [15] mentioned that it was not clear from the included studies whether it was the equipment per se, its maintenance, or the people who used rental equipment that resulted in rental equipment being a risk factor.

Regarding the question of who is renting ski equipment in a professional ski-rental shop, our results revealed that those persons were more likely females, foreign tourists, less-skilled and more cautious skiers. In addition, persons using rented skis showed a significantly lower body height and body weight which might be due to the higher proportion of females in this group.

The elevated ACL injury risk in persons with rented skis might be also partly caused by female sex as in alpine skiing there decisive sex differences in the rates of knee injuries exist, i.e., female recreational skiers have twice the knee injury incidence of male skiers, and the ACL injury risk is three times greater in female skiers [6,24]. This might be on the one hand due to hormonal, anatomical and neuromuscular risk factors which distinguish males from females [25,26], and on the other hand due to potential sex differences in equipment-related factors such as binding adjustment [8,27] or use of rented skis.

Regarding the higher proportion of tourists with rented skis, Ruedl et al. [12] compared in an earlier study a cohort of tourists and locals suffering from a skiing or snowboarding injury where 33% of tourists vs. 8% of Austrian locals rented equipment. There is evidence that skiing experience and skill level in Austrian locals (also due to the alpine environment in Austria and the implementation of winter sport activities in the Austrian physical education curriculum) is on average higher than in winter sport tourists from other countries [12] and that a lower skill level is often associated with a more cautious behavior on ski slopes [28,29]. In line with these results, Johnson et al. [30] pointed out that the injury rate for rental equipment might be higher than that for people skiing their own skis because the former is most often used by less-skilled skiers. In general, skiing injuries are more likely caused by a lower skill level than higher risk-taking behavior [31,32].

### 4.2. Ski Geometry Parameters

Mean absolute and relative ski length of rented skis was significantly lower compared to owned skis. This result for rented skis might be caused by the higher proportion of females with an on average lower body height compared to males, but also with the more than 2-fold higher proportion of less-skilled skiers because for beginners and intermediates performing ski turns is easier with shorter skis.

More-skilled skiers (advanced and experts) in this study used skis with a significantly higher absolute and relative ski length (data not shown) compared to less-skilled skiers. Longer skis allow wider turn radii and thus also higher speeds on ski slopes [33]. However, in the case of a loss of control or a skiing error, longer carving skis can act as a longer lever arm to bend or twist the leg leading to an injury of the ACL [11,34].

Rented skis had a significantly lower mean side-cut radius and significantly lower mean widths of the tip, waist and tail of the ski. The side-cut radius depends on the ski length as well as on the widths of the tip, waist and tail of the ski [11]. Thus, it is not surprising that shorter skis with lower widths have lower side-cut radii. A lower side-cut radius is associated with a higher self-steering effect of the ski [35], i.e., the ski turns itself if the ski is edged and loaded [33]. On the one hand, this makes turning on slopes easier, especially for less-skilled skiers. With a small side-cut radius a skier can perform tight turns with a minimum of skidding, as long as the ski is loaded and under control [33]. On the other hand, however, in uncontrolled situations such as a sudden catching of an edge a ski with a small side-cut radius will carve inward sharply and forces the knee joint into an excessive valgus position and causes a rotation of the lower leg [33]. For instance, the most commonly self-reported ACL injury mechanism in recreational skiing is the forward twisting fall with a valgus external rotation of the knee joint and the main self-reported cause leading to an ACL injury is catching an edge of the carving ski [36,37]. This is of interest regarding the results of the waist-to-tip ratio as rented skis in this study had significantly lower ski width ratios, e.g., a lower waist-to-tip width ratio means that the ski shovel is much wider compared to the waist width. With a broader shovel, it seems easier to catch an edge of the front part of the ski leading to a subsequent fall. In accordance, Ruedl et al. [38] recently found that a lower waist-to-tip width ratio increases ACL injury risk in recreational skiers. Apparently, not only side-cut radius or absolute widths of the skis but also ratios between ski widths seems of preventive interest, especially in less-skilled skiers. However, to evaluate the potential preventive impact of the three different width ratios on self-steering, turning radii and skiing speed, well-designed future studies are needed to establish explanation mechanisms.

### 4.3. Standing Heights and Standing Height Ratio

Mean standing heights of the front and rear components were significantly lower in skiers with rented skis compared to skiers using own skis. In contrast, standing height ratio was significantly higher in rented skis although the mean difference was very small. Senner et al. [35] assumed that an increased standing height causes skidding which could lead to higher strain on the knee joint due to an increase in the lever arm for lateral forces. Therefore, lower standing heights at the front and rear components of the ski binding could be valuable for prevention of knee injuries. On the other hand, too low standing heights could cause an increased impact rate between the ski boot and the snow when performing carved turns, potentially leading to a loss of balance resulting in a subsequent fall with an injury [11].

Recently, Ruedl et al. [11] showed in a case–control study that the standing height ratio was independently associated with an ACL injury. In uninjured skiers, they found a standing height ratio of approximately 89% (similar to plantarflexion rotation of the ski boot) compared to approximately 95% (a more horizontal ski boot) in ACL-injured skiers [11]. An increase in standing height ratio significantly increased ACL injury risk [11]. It could be speculated that a lower standing height ratio (elevated rear component of the ski binding compared to the front component) potentially moves the skier into a more forwarded position. Thereby, the skier’s center of gravity shifts forward which could increase the applied load on the front part of the ski binding potentially allowing a better steering of the skis [11].

To sum up, skiers using rented skis showed a three times higher ACL injury risk in this study. However, so far it is unclear whether this higher risk is caused by individual factors of persons, i.e., female sex and/or lower skill level, who rented skis or by the ski geometry parameters and standing heights of the rented skis. Although a lower ski length and smaller side-cut radius of rented skis could be of some preventive impact, the lower waist-to-tip width ratio and the higher standing-height ratio in rented ski potentially increase injury risk in skiing. Thus, when renting skis in ski-rental shops, especially recreational skiers with a lower skill level should consider these ski geometry parameters by taking skis with a not too broad ski tip shovel and where the rear component of the ski binding is more elevated than the front component.

Furthermore, ski lessons for less-skilled skiers could be useful not only to improve skiing skills [14] but also to become familiar with the slide characteristics of the rented skis.

Regarding equipment as a preventive measure for winter sport injuries, especially for ACL injuries, it seems better using own skis than renting skis. Usually, compared to a busy ski-hire facility more time and personal attention is available to individuals purchasing gear with a wider choice of products fitting their specific size and requirements [18]. However, once new equipment leaves the ski shop after buying, its effectiveness begins to decline due to its use on ski slopes [30]. Thus, skier should have his or her equipment checked by a qualified ski shop at least once per season [30]. On the other hand, using own skis more often during a winter season potentially increases confidence in the equipment and skiing experience and skill level and thus can decrease injury risk on alpine ski slopes [14,39].

### 4.4. Limitations

Due to this type of study, we cannot entirely exclude a potential selection bias or information/measurement bias. Moreover, changes based on the evolution of the equipment during the study period may also have influenced the presented findings. In addition, considering that ACL injuries occur commonly in noncontact situations during direction changes and cutting maneuvers, the incidence of an ACL tear is 2.8–3.5 times greater in females than in males [24,40,41]. However, the large sample size is considered as a major strength of the study.

## 5. Conclusions

In conclusion, skiers with rented skis have a 3-fold higher ACL injury risk in this study. Persons who rented skis in a ski-rental shop were more likely female, smaller and lighter, tourists outside from Austria, reported lower skiing skills and to be more cautious skiers. Ski length, side-cut radius, ski widths and ski widths ratios showed significantly lower values for rented skis. Standing heights of the front and rear components of the ski binding were significantly lower while standing height ratio was significantly higher for rented skis.

Therefore, beside individual factors such as sex and skill level, equipment-related factors as ski length, ski geometry parameters, standing heights and standing height ratio should be considered when renting skis.

## Figures and Tables

**Table 1:** Univariate comparison of individual and ski-geometry-related risk factors between skiers with own skis and with rented skis.

	Own Skis	Rented Skis	*p*-Value
ACL injury (%)			<0.001
Uninjured	84.8 (*n* = 1022)	63.7 (*n* = 366)	
Injured	15.2 (*n* = 183)	36.3 (*n* = 209)	
Sex (%)			<0.001
Male	54.0 (*n* = 651)	45.0 (*n* = 259)	
Female	46.0 (*n* = 554)	55.0 (*n* = 316)	
Age (years)	39.1 ± 13.4 (*n* = 1205)	39.5 ± 12.0 (*n* = 574)	0.185
Body height (cm)	174.0 ± 8.6 (*n* = 1205)	173.2 ± 9.0 (*n* = 575)	0.010
Body weight (kg)	75.3 ± 14.0 (*n* = 1201)	73.4 ± 13.0 (*n* = 575)	0.019
Nationality (%)			<0.001
Austria	37.9 (*n* = 457)	16.5 (*n* = 95)	
Others	62.1 (*n* = 748)	83.5 (*n* = 480)	
Skill level (%)			<0.001
More skilled	80.1 (*n* = 965)	54.1 (*n* = 311)	
Less skilled	19.9 (*n* = 240)	45.9 (*n* = 264)	
Risk-taking behavior (%)			0.032
More cautious	64.3 (*n* = 775)	69.9 (*n* = 400)	
riskier	35.7 (*n* = 430)	30.4 (*n* = 175)	
Ski length (cm)	166.0 ± 9.6 (*n* = 1181)	161.8 ± 9.4 (*n* = 552)	<0.001
Relative ski length (%)	95.4 ± 3.8 (*n* = 1181)	93.5 ± 3.5 (*n* = 552)	<0.001
Side-cut radius (m)	14.6 ± 2.8 (*n* = 1137)	13.8 ± 2.3 (*n* = 536)	<0.001
Tip width (mm)	121.3 ± 8.1 (*n* = 835)	119.5 ± 7.2 (*n* = 212)	0.001
Waist width (mm)	75.8 ± 9.4 (*n* = 1117)	73.3 ± 5.4 (*n* = 439)	<0.001
Tail width (mm)	107.1 ± 8.1 (*n* = 835)	104.8 ± 7.0 (*n* = 212)	<0.001
Waist-to-tip width ratio (%)	63.4 ± 6.3 (*n* = 835)	61.7 ± 5.0 (*n* = 212)	0.001
Waist-to-tail width ratio (%)	71.8 ± 6.5 (*n* = 835)	70.3 ± 5.3 (*n* = 212)	0.004
Tail-to-tip width ratio (%)	88.5 ± 7.3 (*n* = 835)	88.0 ± 7.7 (*n* = 212)	0.042
Standing height front (mm)	40.6 ± 5.6 (*n* = 998)	37.8 ± 5.0 (*n* = 338)	<0.001
Standing height back (mm)	45.5 ± 6.0 (*n* = 999)	42.1 ± 6.1 (*n* = 339)	<0.001
Standing height ratio (%)	89.5 ± 6.7 (*n* = 998)	90.2 ± 7.5 (*n* = 338)	0.030

## Data Availability

Not applicable.
